# Mobile Ecological Momentary Diet Assessment Methods for Behavioral Research: Systematic Review

**DOI:** 10.2196/11170

**Published:** 2018-11-20

**Authors:** Susan M Schembre, Yue Liao, Sydney G O'Connor, Melanie D Hingle, Shu-En Shen, Katarina G Hamoy, Jimi Huh, Genevieve F Dunton, Rick Weiss, Cynthia A Thomson, Carol J Boushey

**Affiliations:** 1 Department of Behavioral Science Division of Cancer Control and Population Sciences The University of Texas MD Anderson Cancer Center Houston, TX United States; 2 Department of Family and Community Medicine College of Medicine-Tucson University of Arizona Tucson, AZ United States; 3 Institute for Health Promotion & Disease Prevention Department of Preventive Medicine University of Southern California Los Angeles, CA United States; 4 Department of Nutritional Sciences College of Agriculture & Life Sciences University of Arizona Tucson, AZ United States; 5 Department of Kinesiology Wiess School of Natural Sciences Rice University Houston, TX United States; 6 Department of Health and Human Performance College of Liberal Arts and Social Sciences University of Houston Houston, TX United States; 7 Viocare, Inc Princeton, NJ United States; 8 Department of Health Promotion Sciences Mel and Enid Zuckerman College of Public Health University of Arizona Tucson, AZ United States; 9 Epidemiology Program University of Hawaii Cancer Center Honolulu, HI United States

**Keywords:** diet surveys, diet records, mobile phone, mobile apps, ecological momentary assessment

## Abstract

**Background:**

New methods for assessing diet in research are being developed to address the limitations of traditional dietary assessment methods. Mobile device–assisted ecological momentary diet assessment (mEMDA) is a new dietary assessment method that has not yet been optimized and has the potential to minimize recall biases and participant burden while maximizing ecological validity. There have been limited efforts to characterize the use of mEMDA in behavioral research settings.

**Objective:**

The aims of this study were to summarize mEMDA protocols used in research to date, to characterize key aspects of these assessment approaches, and to discuss the advantages and disadvantages of mEMDA compared with the traditional dietary assessment methods as well as implications for future mEMDA research.

**Methods:**

Studies that used mobile devices and described mEMDA protocols to assess dietary intake were included. Data were extracted according to Preferred Reporting of Systematic Reviews and Meta-Analyses and Cochrane guidelines and then synthesized narratively.

**Results:**

The review included 20 studies with unique mEMDA protocols. Of these, 50% (10/20) used participant-initiated reports of intake at eating events (event-contingent mEMDA), and 50% (10/20) used researcher-initiated prompts requesting that participants report recent dietary intake (signal-contingent mEMDA). A majority of the study protocols (60%, 12/20) enabled participants to use mobile phones to report dietary data. Event-contingent mEMDA protocols most commonly assessed diet in real time, used dietary records for data collection (60%, 6/10), and provided estimates of energy and nutrient intake (60%, 6/10). All signal-contingent mEMDA protocols used a near real-time recall approach with unannounced (ie, random) abbreviated diet surveys. Most signal-contingent protocols (70%, 7/10) assessed the frequency with which (targeted) foods or food groups were consumed. Relatively few (30%, 6/20) studies compared mEMDA with the traditional dietary assessment methods.

**Conclusions:**

This review demonstrates that mEMDA has the potential to reduce participant burden and recall bias, thus advancing the field beyond current dietary assessment methods while maximizing ecological validity.

## Introduction

Diet plays significant direct and indirect roles in the etiology and prevention of chronic diseases including type 2 diabetes, coronary heart disease, cancer, and obesity [1]. It has been estimated that an unhealthy diet was the leading cause of premature death in the United States, contributing to more than 500,000 deaths in 2016 [1]. Despite these statistics, we lack a clear understanding of how patterns of dietary intake affect health through the life span because of dietary measurement limitations. Current dietary assessment methods including 24-hour dietary recalls, food frequency questionnaires, and dietary records have advantages and disadvantages in research settings [2]. The 24-hour dietary recalls are considered the gold standard in dietary assessment because they produce the highest quality data, but they rely heavily on participant memory and can require the greatest amount of time to acquire when administered by the interviewer. Food frequency questionnaires are more easily administered and provide good estimates of patterns of intake; however, they are also subject to participant memory and are most likely to underestimate energy intake because of limitations including a lack of cultural tailoring or limited food lists. Diet records or diet diaries minimize reliance on memory when foods and beverages are recorded when consumed; however, participants require a high level of motivation as well as training to improve recording accuracy. Furthermore, research shows that people pay little attention to when and what they eat [3], and factors such as age, sex, and weight status can influence the accurate recall of food and estimation of portion size [2,4-8]. Finally, methods that do not use unannounced recalls (eg, diet records) may be prone to biases such as social desirability or reactivity bias, which may lead to participants underreporting or omitting of foods or beverages consumed or changing their usual dietary behaviors because of the awareness that they are being observed [9]. Such errors in reporting are known to create conflicting evidence linking diet to health outcomes that could be addressed if we could more robustly measure diet [10,11]. Furthering our understanding of the connection between diet and disease will require improvements in the dietary assessment methodologies. For this reason, the research community has recognized the need for new dietary assessment methods that can reduce misreporting and recall biases [12,13].

Recent advancements in digital technology and computational sciences have laid the foundation for emerging dietary assessment solutions. These advancements have catalyzed the development of new methods aimed at automating the assessment of dietary intake, thereby limiting or eliminating the need for self-report. In particular, 2 such new dietary assessment methods have been developed: image-based dietary assessment [14-17] and the detection of food intake by biomechanical sensors [18-29]. Single image-based assessment methods use photos of foods and beverages along with fiduciary markers before and after consumption. The time-stamped images are either reviewed and coded into nutrition software by trained research staff (image-assisted assessment) or are analyzed by software designed to identify the type and volume of foods in the image (image-based assessment). Alternatively, approaches using gyroscopes, microphones, and mechanical or electrical impedance sensors have been integrated into wearable devices such as watches and headsets or are designed to be mounted on teeth to detect wrist or hand motion or patterns of chewing or swallowing indicative of food intake (eg, number of bites). However, the automation of dietary assessment using these mobile-based approaches remains in the proof-of-concept stage. There is a lack of large-scale validation studies demonstrating their utility to assess dietary intake in community-dwelling populations. For instance, the mean detection accuracy of image detection and wearable devices has been acceptable in controlled, laboratory settings (range 73%-99%) [19,21,23,24,26-35], but limited testing has been done in natural settings. The use of mechanical sensors in research is further hindered by poor battery life, having to remember to wear or use the device, needing to turn the device on or off to avoid detection errors, and the conspicuousness or general discomfort of having to wear collars, wires, or harness accessories. Substantial work will be needed before these methods can accurately quantify energy or nutrient intake for research purposes. Therefore, novel dietary assessment methods addressing limitations of the traditional dietary assessment methods and methods that bridge the gap between traditional and newer methods of dietary assessment are needed [12,13].

Another less developed dietary assessment method with the potential to improve the validity and reliability of dietary assessment is the mobile device–assisted ecological momentary assessment (mEMA). mEMA is based on the foundation of ecological momentary assessment (EMA) described by Shiffman et al in 2008 [36]. EMA involves the repeated sampling of a person’s current behaviors and experiences in real time, in their natural environments. Currently, there are 2 mobile device–assisted ecological momentary diet assessment (mEMDA) approaches: event-contingent mEMDA and signal-contingent mEMDA. Event-contingent mEMDA most often occurs in real time at the time of eating (or drinking). The frequency of sampling is determined by the number of times a participant reports eating. Here, the act of initiating a meal or snack triggers either the real-time recording of dietary intake (eg, dietary records or image-assisted dietary records). The advantage of real-time diet records is that they are intended to capture all foods and beverages consumed without having to recall the events at a later time. Although this is an advantage, the key limitation of event-contingent mEMDA is that there are no unannounced sampling events. The self-monitoring of dietary intake can be influenced by psychosocial and behavioral factors that introduce reactivity and measurement bias including eating behaviors (dietary restraint or disinhibition), social desirability, body image, or depression and anxiety [5]. Furthermore, with image-assisted diet records, there is the potential for data entry bias by research staff viewing images or for intentional or unintentional reporting errors (eg, inaccurate report of portion sizes) by participants, particularly if foods are omitted from the images or the images are not taken at multiple points of a meal (eg, before and after a meal).

Signal-contingent mEMDA relies on researcher-initiated, signaled prompts to participants that trigger the recall of current or recent dietary intake. Although study participants are often prompted multiple times per day, signal-contingent mEMDA does not always allow for the real-time assessment of dietary intake. Rather, assessment surveys often include questions referencing dietary intake occurring within the most recent interval of time (eg, past 30 min). Moreover, study participants are most often asked to report the consumption of specific foods or foods from specified food groups (eg, fruits and vegetables) by means of a brief survey. The frequency of sampling using signal-contingent mEMDA is determined by the researcher and can occur randomly at fixed or semifixed times or randomly within fixed or semifixed time intervals. As with image-assisted or image-based dietary records, these momentary dietary assessments can be time-stamped and are either stored or transmitted for later database integration. Although this method benefits from random (unannounced) sampling, short recall intervals, and reduced participant burden, the commonly used sampling schemes, limited study durations, or limited food lists can hinder the quantification of energy or nutrient intakes.

Due to recent advancements made in mobile device hardware and software and the pervasive use of mobile devices, EMA-based methods leverage the capabilities of mobile technology, offering researchers an opportunity to assess the dietary intake of study participants as they are occurring in natural settings. Both event-contingent and signal-contingent mEMDA seek to reduce recall bias and thereby improve the accuracy of dietary assessment by eliminating or shortening the recall interval and reducing participant burden while maximizing ecological validity as compared with the traditional dietary approaches. However, neither approach has been well characterized nor adequately compared against objective biomarkers or other methods of energy intake assessment (eg, 24-hour dietary recalls). Focused efforts are needed to develop mEMDA methods for their consistent and replicable application in research settings. Therefore, the goals of this systematic review were to summarize the event-contingent and signal-contingent mEMDA protocols that have been used in research to date, to characterize key aspects of these assessment approaches (eg, design, data collection, data processing and dietary analysis, and dietary outcomes), and to discuss the advantages and disadvantages of each as well as implications for future mEMDA research. The focus of the review was on studies summarizing unique dietary assessment protocols using mobile devices to facilitate event-contingent or signal-contingent EMA to assess dietary intake.

## Methods

### Literature Search

A systematic strategy was devised by 6 authors (SMS, YL, MDH, JH, GFD, CAT, and CJB) to search the MEDLINE, EMBASE, PubMed, PsycINFO, and IEEE explore databases for all relevant literature published through February 2018. The search was limited to articles written in the English language and conducted with humans. The database search included the use of controlled vocabulary and keywords to identify studies addressing dietary assessment, mobile devices, and ecological momentary assessment. Keywords such as “nutrition assessment,” “diet surveys,” “diet records,” “energy intake,” “meals,” or “eating” combined with “text messaging,” “mobile phone,” “mobile applications,” “micro-electrical-mechanical systems,” or “wearable electronic devices” were included as MeSH search terms. In addition, non-MeSH search terms were included to be complete: “caloric intake,” “food diary,” “diet monitoring,” “food tracking,” “diet tracking,” “diet assessment,” or “calorie tracking” and “text messages,” “cell phone,” “smartphone,” “tablet computer,” “mobile health,” “eHealth,” “mHealth,” “digital health,” “mobile technology,” or “experience sampling.” Search terms synonymous with the terms above that did not produce additional references (eg, “food record”) were omitted from the final search conducted by the author (SMS). References cited in all included studies and studies citing included studies (referred to as “other sources” in the Preferred Reporting of Systematic Reviews and Meta-Analyses [PRISMA] diagram) were also reviewed to identify any additional studies to be screened for inclusion.

### Study Inclusion and Exclusion Criteria

Eligible studies had protocols using mobile devices and event-contingent or signal-contingent EMA approaches to assess dietary intake in research settings. These included diet assessment studies as well as behavioral trials where dietary intake was assessed. Dietary intake was defined as the quantification of energy intake, macro- or micronutrients, discrete foods, servings, or food groups. Literature returned by the search was screened first by article type then by title, abstract, and the described methods by 2 authors (SMS and YL). Only original research articles were included. Abstracts, review papers, editorials, etc, were excluded. Additionally, studies were excluded if the title, abstract, or methods indicated (1) the study was not diet-related (eg, nondiet-related papers, proof-of-concept, or technology design papers); (2) the studies were interventions with non-EMA dietary assessment methods (24-hour dietary recalls and food frequency questionnaires); (3) did not assess dietary intake (eg, binge eating lapses, availability of snack foods, and food craving); (4) used self-monitoring approaches without dietary analysis; (5) were described in an earlier study or were considered a secondary analysis; or (6) were not peer-reviewed journal articles (eg, abstracts, editorials, discussions, evaluations, reviews, reports, news, notes, surveys, or content analysis). Additional papers referencing the included studies were used to obtain methodological details not otherwise provided in the included studies.

### Data Extraction and Analysis

Data were extracted into a structured coding form according to PRISMA guidelines [37] and the Cochrane Handbook for Systematic Reviews of Interventions [38]. A data extraction form developed for this review was used by 2 authors (SES and KGH) to independently extract and review characteristics from all studies. Extracted data represented details on mEMDA protocols and included but were not limited to (1) mobile platform, (2) sampling duration, (3) prompt approach (signal-contingent only), (4) prompt frequency (signal-contingent only), (5) data collection method, (6) data processing and nutrient analysis, (7) diet data outcomes, and (8) protocol adherence. A comprehensive list of data extracted from included studies is provided as Multimedia Appendix 1. Discrepancies in the extracted data were resolved by a discussion between 2 expert reviewers (SMS and YL) to complete the dataset. In several cases, studies closely related to the included studies were reviewed for additional information to resolve issues of missing or unclear data. Extracted data were descriptive in nature. The data were synthesized narratively and tabularized with the intent of summarizing available protocols for assessing diet using mobile EMA methods.

## Results

### Literature Search

**Figure 1:**
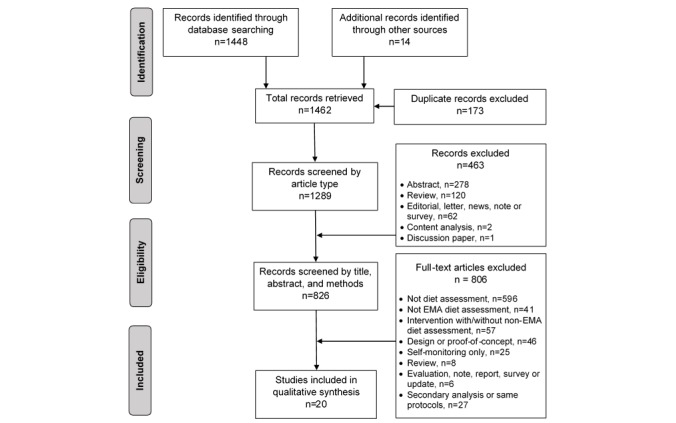
Preferred Reporting of Systematic Reviews and Meta-Analyses diagram. EMA: ecological momentary assessment.

The literature search yielded 1462 studies, of which 173 were duplicates, leaving 1289 articles to be screened for eligibility. A total of 463 articles were excluded based on an initial screening indicating these were not journal articles. Thus, 826 articles were screened by title, abstract, and methods for eligibility. After 806 articles that did not meet the inclusion criteria were excluded, 20 studies were included in the review (see PRISMA diagram, Figure 1). Among the 20 studies included in the review, 10 used event-contingent mEMDA protocols and 10 used signal-contingent mEMDA protocols to assess dietary intake. An additional 19 related journal articles were used to obtain methodological details not provided in the included studies. Of the 20 included studies, 7 were behavioral trials where dietary intake was assessed. The remaining 13 were diet assessment studies.

### Summary of Event-Contingent Studies

The protocols in studies using event-contingent mEMDA are summarized in Table 1. A total of 10 studies used event-contingent mEMDA protocols in nutrition-related research [39-48]. Additional details about protocols used in the included studies that were not described fully were extracted from related journal articles that used the same mEMDA protocol [30,49-60].

**Table 1:** Event-contingent, mobile ecological momentary dietary assessment.

First author, year	Mobile platform and device	Sample period	Data collection^a^	Data processing and nutrient analysis	Diet data outcomes
Ashman et al, 2017 [39]	Internet-based, mobile phone app	3 days	Image-assisted dietary record: images taken before and after meals with fiducial marker	Dietitians analyzed food images with FoodWorks software (The Nutrition Company)	Energy, protein, dietary fat, carbohydrates, and select micronutrients
Boushey et al, 2017 [40]	Mobile phone app	7.5 days	Image-assisted dietary record: images taken before and after meals with fiducial marker	Trained analysts analyzed food images with Food and Nutrient Database for Dietary Studies (United States Department of Agriculture)	Energy intake
Della-Torre et al, 2017 [41]	Internet-based, mobile phone app	4 days	Dietary record: food and beverages chosen from 900 options	Automated app output (study-specific food composition database)	Energy, protein, dietary fat, carbohydrate, fruit and vegetables, and dairy
Grenard et al, 2013 [42]	PDA^b^ device and software	7 days	Dietary record: food and beverages chosen from 3 groups	Data downloaded from PDA by researchers (no nutrient database used)	Number of sweetened drinks, sweet snacks, salty snacks, and sweet or salty snacks
Hingle et al, 2013 [43]	Social media (mobile phone app; Twitter)	3 days	Dietary record: food and beverages chosen from 24 groups	Web-based data capture app (ViBE) used to automatically calculate output (no nutrient database used)	Number of times each food category was reported
Martin et al, 2012 [44]	Mobile phone app	6 days	Image-assisted dietary record: images taken before meals with fiducial marker	Image analysis by 2-step process: human raters and computer automation with Food and Nutrient Database for Dietary Studies (United States Department of Agriculture)	Energy, protein, dietary fat, carbohydrates, and select micronutrients
Schuz et al, 2015 [45]	Mobile phone app	10 days	Dietary record: items labeled as breakfast, lunch, dinner, snacks, and drinks	Data downloaded from app by researchers (no nutrient database used)	Frequency of meals, snacks, nonalcoholic drinks, or alcoholic drinks
Seto et al, 2016 [46]	Mobile phone	6 days	Voice-annotated video with time stamp	Dietitians analyzed the videos and coded the portion size and food groups (no nutrient database used)	Portions of total meal, dairy, protein, grains, vegetables, and fruits
Thomas et al, 2011 [47]	PDA device and software	6 days	Dietary record: food and beverages chosen from 8 groups with manual entry of food type and portion size	Data downloaded from PDA by researchers (no nutrient database used)	Food group servings
Waki et al, 2014 [48]	Mobile phone app	3 months	Image-assisted dietary record: images taken before meals	Automatic photo processing by study-specific software and Dietary Reference Intakes	Energy, protein, dietary fat, carbohydrate, dietary fiber, and sodium

^a^All food and beverage recorded unless otherwise noted.

^b^PDA: personal digital assistant.

#### Design

Of the 10 studies, 5 were mobile phone–based [40,44-46,48]: 3 used mobile phone apps [40,44,48] and 2 used the mobile phone camera function [44,46]. The remaining 5 studies used a PDA device with customized software [42,47], an internet-based app [39,41], or social media (Twitter) [43]. All studies assessed dietary intake continuously throughout each day. The sampling duration ranged from 3 days to 3 months, with 6 days being the most common.

#### Data Collection Methods

A total of 6 studies collected data by dietary records [39,41-43,45,47]. A note-taking app with image capture was used in 1 study [39]. Others had study participants record the consumption of predefined food types or food groups [41-43,45,47]. Participants were asked to take photos of all food and beverage consumed without additional note taking in 3 studies [40,44,48], and 1 study collected dietary data by voice-annotated video taken with a mobile phone [46]. In 1 study, all food and beverages were provided to participants to take home during the study period and encouraged them to supplement with usual foods and beverages not provided [40]. All other studies collected dietary data based on a participant’s usual eating behaviors. Hingle et al [43] collected 1756 food-related hashtags via Twitter across all participants over 3 days. In Seto et al’s [46] 6-day study, 72 food items were reported via video per participant. Participants on average reported 7 food entries via dietary record per day in Ashman et al’s 3-day study [39]. Alternative methods to capture missed meals (ie, pen and pencil or voice recording) were used in 2 studies. EMA prompts were used at standard or usual breakfast, lunch, dinner, and snack times as a reminder to log eating events in 1 study [44].

#### Dietary Analysis and Outcomes

Trained dietitians or research staff were involved in 5 studies to analyze the data based on a food composition database or similar software [39-41,44,46]; 3 studies downloaded data from the mobile device to perform further data analysis without the use of a nutrient database or software [42,45,47]; these studies assessed frequency or servings of food intake. Output automatically generated by a non-nutrient-related software or app was used in 2 studies [41,43]; 1 study estimated intake of nutrients, energy, and food groups [41], the other assessed frequency of food category [43]. The nutrient analysis was automated within the study app in 1 study [48]. Participants received feedback about nutritional balance and energy balance of the meal as well as advice on dietary modification within the app. With respect to the primary outcomes assessed, 5 studies estimated energy intake [39,40,44,45,48]. Macro- or micronutrients were estimated in 4 studies [39,40,44,48]. Portions or servings consumed from designated food groups were estimated in 2 studies [46,47]. Dietary data at within-day level (ie, for each meal) were provided in 4 studies [43,45,46,48]; the remaining studies provided dietary data summary at the day level.

#### Protocol Adherence

Data regarding adherence to the dietary data collection protocols described in the studies (eg, reporting all eating events) were not provided in any of the event-contingent studies. This was most likely because of not having objective knowledge of when eating actually occurred. However, 3 studies reported the number of eating events captured or frequency with which foods were consumed [39,43,46].

#### Comparison Testing

There were 4 studies comparing their mEMDA approach against a traditional dietary assessment method [39-41,44]. In 2 studies, estimated energy intake was compared with doubly labeled water [41,44]. Martin et al [44] found no significant difference in energy intake between the estimation from their mEMDA approach (Remote Food Photography Method) versus the doubly labeled water in a sample of overweight and obese adults (−152 ± 694 kcal/day, *P*=.16). However, in another comparison test related to the mEMDA method used by Martin et al, Nicklas et al [50] found the Remote Food Photography Method underestimated energy intake when compared with doubly labeled water by an average of 222 kcal/day (−15.6%, *P*<.001) in a sample of minority (Hispanic and African American) preschool children (data reported by their caregivers). Boushey’s study [40] aimed to test the accuracy of the estimated energy intake from the mobile Food Record (mFR) against energy expenditure assessed by doubly labeled water in a community sample of 45 adults aged 21-65 years. On the basis of the comparison, the mean percent of underreporting on the mFR was 12% (SD 11) for men and 10% (SD 10) for women. Estimated intake to 24-hour dietary recalls was compared in 2 studies [39,41]. Della-Torre et al [41] developed and evaluated an electronic mobile-based food record, electronic carnet alimentaire (e-CA) for a research setting. They evaluated e-CA’s accuracy in terms of energy, macronutrient, and food group intake in a convenience sample of 21 adults and found the primary diet data had more than 85% agreement with the 24-hour dietary recall. Ashman et al [39] evaluated relative validity of the DietBytes image-based dietary assessment method for assessing energy and nutrient intakes in 25 pregnant women and found the macronutrient and energy intake had more than 90% agreement with the 24-hour dietary recall.

### Summary of Signal-Contingent Studies

The protocols in articles that only used signal-contingent mEMDA approaches are summarized in Table 2. A total of 10 studies described signal-contingent mEMDA used in nutrition-related research [61-70]. Additional details were extracted from multiple related journal articles [66,71-76].

#### Design

Of the 10 studies, prompts were delivered via mobile phone in 7 studies [61-63,66,67,69,70]. Of these 7 studies, 5 used mobile apps [62,63,66,67,69], 1 used short message service text messaging [61], and 1 used a Web-based survey [70]. A wrist-worn electronic diary device was used in 2 other studies [64,65], and another study used an iPod Touch [68].

Of the 10 studies, 5 studies used *random* intervals for prompting [62-64,69,70] with frequencies ranging from 3 to 10 prompts per day. Of these 5 studies, 3 studies assessed dietary intake “since the last prompt” at varied time intervals [64,69,70], 1 study assessed dietary intake in the past 2 hours [63], and 1 study assessed dietary intake in real time [62].

The other 5 studies prompted surveys at *fixed* intervals [61,65-68], and frequencies ranged from 4 to 14 prompts per day. Of these 5 studies, 2 studies assessed dietary intake “since the last prompt” at varied time intervals [66,68], 2 studies assessed dietary intake in the past 1-3.5 hours [65,67], and 1 study assessed dietary intake in real time [61]. The sampling duration for the 10 studies ranged from 4 days to 6 weeks, with the most common duration being 7 days (n=4).

**Table 2:** Signal-contingent, mobile ecological momentary dietary assessment.

First author, year	Mobile platform	Sample period	Prompt approach	Prompt frequency (recall interval)	Diet data collection (format, source)	Diet data output outcomes (units)
Berkman et al, 2014 [61]	Mobile phone SMS^a^ text messages	14 days	Individualized fixed time	4 prompts: 3 real time, 1 retrospective (since last prompt)	1 survey item (open-ended, preselected snack food)	Frequency of snack intake
Bruening et al, 2016 [62]	Mobile phone app	4 days	Random interval	8 prompts: 7 real time, 1 retrospective prompt (past 3 hours)	2 survey items (multiple choice, 8 food groups, and 8 beverage groups)	Bread or grains, entrée, fruit and vegetables, salty foods, and sweets intake (number and percent of prompts)
Dunton et al, 2015 [63]	Mobile phone app	8 days	Random interval	Mother: 4 or 8 retrospective prompts (past 2 hours); Child: 3 or 7 retrospective prompts (past 2 hours)	1 survey item (multiple choice, 5 food groups)	Healthy and unhealthy eating (frequency of prompts)
Miller et al, 2016 [64]	Wrist-worn electronic diary	6 weeks	Random interval	3 retrospective prompts (since last prompt)	1 survey item (open-ended)	Low glycemic index foods (servings)
Powell et al, 2017 [65]	Wrist-worn electronic diary	7 days	Fixed time (±10 min)	14 retrospective prompts (past hour)	8 survey items (8 food groups, yes or no)	Snack and fruit and vegetable intake (ranked portion sizes)
Richard et al, 2017 [66]	Mobile phone app	7 days	Fixed time	5 retrospective prompts (since last prompt)	1 survey item (open-ended)	Snack intake density (kcal/100 g)
Spook et al, 2013 [67]	Mobile phone app	7 days	Fixed time (±30 min)	5 retrospective prompts (past 3.5 hours)	3 survey items (multiple choice and visual analog scales, 3 food groups)	Number and frequency of snack, fruit and vegetable, and soda intake
Strahler and Nater, 2018 [68]	iPod Touch app	4 days	Fixed time	5 retrospective prompts (since last prompt)	3 survey items (multiple choice recoded to yes or no)	Frequency of meal type, main component, and drink consumption
Wouters et al, 2016 [69]	Mobile phone app	4 days	Quasi-random interval (average 90 min)	10 retrospective prompts (since last prompt)	Digital food log of snacks (open ended)	Energy intake carbohydrate, fat, and protein
Zenk et al, 2014 [70]	Mobile phone Web-based survey	7 days	Random interval	5 retrospective prompts (since last prompt)	9 Web-based survey items (9 food groups, yes or no)	Number of snacks consumed (0 or more than 1)

^a^SMS: short message service.

#### Data Collection Method

All studies used an abbreviated survey format to collect dietary data. The number of diet-related survey items ranged from 1 to 9 items. There were 3 studies choosing their dietary variables from intake patterns specific to the targeted population [62,67,70]. A search function linked with a national food composition database within the study app was provided in 1 study [69]. The number of overall diet-related survey items ranged from 1 to 16 items. Five studies used multiple choice options for participants to answer the survey [62,63,67,68,70]; 1 study used yes/no choice [65]; 1 study asked participants to enter number of servings [64]; 1 study asked participants to enter specific study codes that indicate servings, craving, and hunger [61]; 1 study used free text to record snacks [66]; and 1 study asked participants to record intake by searching the food database within the app [69].

#### Dietary Analysis and Outcomes

Response data were downloaded from the respective mobile platform by researchers to perform analysis without the use of a nutrient database for all studies. There were 7 signal-contingent studies reporting on the occurrence or frequency of (targeted) food or food group intakes at the day-level that were most relevant to the research [61-63,65,67,68,70]. Three studies focused on snack intake only [65,66,70]. Only 2 studies estimated energy intake [66,69]. Another study estimated servings of low glycemic index foods [64].

#### Protocol Adherence

EMA prompt response rate was reported in 8 of the 10 studies [61,63,64,66-70]. Response rates ranged from 23%-63% per day to 98% across the study period. The mean response rate across all studies was 79% with a median of 74%.

#### Comparison Testing

There were 2 studies comparing their mEMDA protocols against 24-hour dietary recalls [62,63]. In Bruening’s study [62], participants completed 3 days of dietary recalls (2 weekdays and 1 weekend day). Each food item reported in the dietary recalls was coded to match the food groups used in the mEMDA protocol. The concordance rate at the day level ranged from 79% for entrees to 94% for fruit and vegetables in a sample of college students. In Dunton’s study [63], children completed 2 days of dietary recalls. Each food item reported in the dietary recalls was coded to match the food groups used in the mEMDA protocol. Furthermore, time of food intake from the dietary recalls was matched with the 2-hour recall time windows that were used in the mEMDA protocol. The 2-hour concordance rate ranged from 65% for fruits/vegetables to 90% for soda/energy drinks in a sample of children (mean age=10 years) [74].

## Discussion

### Summary of Key Findings

This systematic review summarized the existing protocols for measuring dietary intake using 2 mEMDA approaches: event-contingent and signal-contingent mEMDA. Studies describing 20 unique mEMDA protocols were included in the review. Half of the studies used event-contingent mEMDA protocols and half used signal-contingent mEMDA protocols. Most studies used mobile phones to collect dietary data. Studies that used event-contingent mEMDA most commonly assessed diet in real time, used dietary records to collect data, and provided estimates of energy and nutrient intake for data collection purposes. All signal-contingent mEMDA studies used near real–time recalls and unannounced abbreviated diet surveys and assessed the frequency of consumption of foods or food groups most relevant to the research. Only 6 (30%) mEMDA studies directly compared mEMDA and dietary outcomes measured by the traditional dietary assessment methods (eg, 24-hour dietary recalls). As such, the evolving body of literature identified in this review supports the application of mEMDA as the next step for advancement in the field of dietary assessment, bridging the gap between traditional methods and newer, more technologically advanced methods (ie, biomechanical sensing and image-based food detection), which are currently under development.

### Key Strengths and Limitations of Mobile Device–Assisted Ecological Momentary Diet Assessment

The mEMDA approaches described in this review have strengths and limitations potentially impacting the quality of estimated dietary intake. Event-contingent mEMDA protocols have several strengths based primarily on the fact that food (and beverage) consumption generally occurs as a discrete event; it serves as a cue to record intake [77]. These studies most commonly assessed diet in real time (eg, at the time of consumption) or used image-assisted or image-based dietary assessment methods to reduce recall bias (eg, dependence on memory) when intake was reported at a later time. Moreover, when integrated with food composition databases or similar software, event-contingent mEMDA protocols most often facilitate the calculation of or automatically calculated estimates of energy and nutrient intake. Finally, the mobile platform of event-contingent mEMDA enables eating events to be time-stamped. This removes the time-keeping burden from participants, allows for better specificity for eating occasions, and enhances the ability to examine the distribution and frequency of eating events across days or weeks [77]. There are also several limitations inherent to event-contingent mEMDA that are equally problematic as the similar traditional methods of dietary assessment (eg, written food records). First, event-contingent mEMDA requires participants to initiate the self-reporting of each eating event over the study period, which may be perceived as burdensome and may lead to omitted data when participants forget or decline to report [36]. Second, the routine anticipation of self-report of dietary intake (vs unannounced recalls) consistent with event-contingent mEMDA is more likely to be biased by psychological reactance or social desirability, whereby people change their usual eating behaviors or intentionally misreport intake so as to not be judged for making diet-related decisions perceived by the individual to be less healthful [5]. Finally, compared with traditional dietary assessment methods, event-contingent mEMDA may be limited by existing on a mobile platform, which might be less acceptable to some populations including people who are less comfortable with technology (eg, older adults) [78] or have low electronic health literacy [79]. For these individuals, written records might be preferred. In addition, compared with signal-contingent mEMDA protocols, which typically employ an abbreviated survey, event-contingent mEMDA more often involves more high-resource data processing to process raw video, photos, or sound files into analyzable food group or nutrient data at meal level or day level requiring software that might not be readily accessible to researchers. Overall, event-contingent mEMDA appears useful for capturing individuals’ intake as it occurs, by eliciting a time-stamped log of all eating events and their contents; however, limitations include greater participant burden (ie, recording all food and beverage consumed and remembering to do so), the increased likelihood of psychological reactance or social desirability biases, and lower acceptance levels in some populations.

The remaining studies (n=10) used signal-contingent mEMDA sampling. All the signal-contingent mEMDA studies used a near real–time recall approach with abbreviated diet surveys, whereas some also incorporated real-time prompting. Most signal-contingent studies assessed the frequency with which foods or food groups most relevant to the research were consumed. Strengths of signal-contingent mEMDA include lower participant burden related to survey brevity and unannounced sampling, which provides a random sampling of eating events throughout the day. Here, participants receive prompts to report their recent intake throughout the day, providing a representative sample of overall daily intake without having to proactively input details about each eating occasion as they occur. Additionally, the majority of existing signal-contingent studies used simplified reporting methods, asking participants whether or not they consumed certain target foods within a recent interval of time (eg, past 30 min or past 2 hours). Though signal-contingent mEMDA has several strengths, there are also limitations to note. First, it may be subject to incomplete data, particularly when sampling windows do not cover the entire day. As a result, some eating events may be omitted. For example, when a meal occurs outside the daily sampling window, such as very early or late in the day, participants may not be prompted to report it. Second, dietary intake captured by signal-contingent mEMDA versus 24-hour recalls is subject to lower specificity of timing, particularly when the recall window is longer (eg, >2 hours), as participants are typically asked to report whether or not food (or beverage) consumption has occurred, without elaboration about the specific time it occurred. Finally, although existing signal-contingent methods have typically used lower resource processing methods (eg, not requiring advanced training or specialized software), the resulting data may be limited to quantifying frequencies of intake as opposed to estimates of energy intake or nutrients due, in part, to the brevity of the surveys, a key aspect of signal-contingent mEMDA. Although survey brevity (and frequency of administration) is important for maintaining high response rates [80], it poses limitations to data quality with regard to how the data are summarized and the extent to which they can be used to describe dietary intake. Future research will be needed to determine how best to balance response rates with the collection of high-quality diet data. To summarize, signal-contingent mEMDA is able to capture a representative (ie, randomly selected) sample of individuals’ daily intake while minimizing the participant burden associated with participant-initiated reporting; however, it may not be suited for time-stamping eating events or quantifying energy or nutrient intakes.

Another limitation of mEMDA methods is that few studies have compared mEMDA protocols with the traditional dietary assessment methods. However, the few that have conducted comparison studies have generally found acceptable agreement in estimated energy intakes and reported concordance. For instance, 2 studies that compared mEMDA reports of estimated energy intake against doubly labeled water [40,44] found that mEMDA underestimated energy intake by an average of 222 kcal/day (−15.6%) [50] and approximately 11% [40], respectively. In 4 studies event-contingent [39,41] and signal-contingent recalls [62,63] mEMDA estimated intake was compared with 24-hour dietary recalls. The 2 event-contingent studies demonstrated 85% agreement for primary diet data [41] and 90% agreement for macronutrient and energy intake [39] when compared with 24-hour dietary recalls. The 2 signal-contingent studies found day-level concordance between foods and beverages reported by mEMDA and next day 24-hour dietary recalls ranged from 79% (entrees) to 94% (fruit and vegetables) in a sample of college students [62] and 2-hour concordance ranged from 65% (fruits and vegetables) to 90% (soda and energy drinks) for children [63]. Similar magnitudes of difference have been reported when comparing traditional methods of dietary assessment (eg, Web- and computer-based 24-hour dietary recalls) with a more objective reference method (eg, direct dietary observation, doubly labeled water, and controlled-feeding studies) [81]; however, it is difficult to determine the validity of any method of dietary assessment because of the inherent measurement errors that obscure differences between the observed and the true reports. Moreover, it is important to note that these studies did not report compliance rates for mEMDA versus the comparison method. Despite these limitations, the studies highlighted in this review demonstrate the feasibility and preliminary evidence of accuracy of mEMDA in research settings. It will be important for developing mEMDA methods to conduct similar comparison studies against more objective reference methods and to compare their protocol adherence rates with the traditional methods of dietary assessment.

### Strengths and Limitations of This Review

Our systematic review is the first to summarize the existing literature of mEMDA for the measurement of diet in research studies and to discuss corresponding strengths and weaknesses. This study was based on a comprehensive search across multiple domains (images, biomechanical approaches, EMA, etc) using current guidance for a robust systematic review process. This study was limited in breadth as a result of many burgeoning methods not yet being applied in research settings and were, therefore, excluded from this review. Furthermore, senior reviewers made final decisions about study inclusion rather than an independent expert, which may have introduced some selection bias. In addition, because of the wide divergence of study measures and reporting of relevant items (eg, protocol adherence), we were only able to narratively describe data collection methods. Furthermore, this review was limited in its ability to describe how accurately mEMDA methods capture diet compared with traditional methods of dietary assessment because of limited reports of such comparison statistics in the existing studies.

### Implications for Future Research

Currently, there is a need within the field of momentary diet assessment to maximize data quality while minimizing participant burden. Ecologically valid and reliable data on individuals’ dietary intake are essential to understand the role of diet on human health through the life span. Due to the known limitations of the existing dietary assessment methods, the research community is motivated to develop new solutions aimed at (semi)automating the assessment of dietary intake. Although the automated methods of real-time image-based detection and real-time detection of food intake by biomechanical sensors or hand-held devices have seen significant progress in terms of identifying foods and estimating portion sizes (detecting wrist or hand motion or patterns of chewing or swallowing indicative of food intake), the design and proof-of-concept data suggest the automation of dietary assessment remains out of reach for the time being. Given these limitations, mEMDA, characterized by the repeated assessment of an individual’s behaviors and experiences in real time or near-real time in their natural settings, represents a novel dietary assessment method. By reviewing the literature and identifying key patterns, strengths, and weaknesses of the existing momentary diet assessment methods, a unique topic of high relevance in the dietary assessment community and EMA community, researchers may better understand and move forward with improving and incorporating mEMDA into their own research. Compared with the traditional methods, mEMDA may reduce participant burden and recall biases while maximizing ecological validity. Therefore, mEMDA has the potential to bridge the gap between currently available methods (eg, 24-hour dietary recall) and newer methods (eg, biomechanical sensors), which are currently under development.

The strengths of event-contingent mEMDA (eg, ability to capture the full day of dietary intake and estimate energy and nutrient intakes) and of signal-contingent mEMDA (eg, lower participant burden and unannounced prompting schemes) could potentially be leveraged to design novel mEMDA methods that reduce their individual limitations. On the basis of these early studies, efforts now need to be focused on standardizing mEMDA methods with the goal of maximizing dietary data quality and ecological validity while minimizing participant burden. With improved standardization, it is likely that validated mEMDA tools will become more widely available to researchers in the future as most methods have been study-specific. Existing studies illustrate the wide range of dietary outcomes assessed through mEMDA methods, and although the ability to develop and tailor assessment items based on a particular study’s needs is an advantage, the divergence of outcome measures and lack of validation remains a major challenge.

## Appendix

Multimedia Appendix 1 Extracted data list.

## Abbreviations

e-CA: electronic carnet alimentaire
EMA: ecological momentary assessment
mEMA: mobile device–assisted ecological momentary assessment
mEMDA: mobile device–assisted ecological momentary diet assessment
mFR: mobile Food Record
PDA: personal digital assistant
PRISMA: Preferred Reporting of Systematic Reviews and Meta-Analyses

## References

[1] Mokdad AH, Ballestros K, Echko M, Glenn S, Olsen HE, Mullany E, Lee A, Khan AR, Ahmadi A, Ferrari AJ, Kasaeian A, Werdecker A, Carter A, Zipkin B, Sartorius B, Serdar B, Sykes BL, Troeger C, Fitzmaurice C, Rehm CD, Santomauro D, Kim D, Colombara D, Schwebel DC, Tsoi D, Kolte D, Nsoesie E, Nichols E, Oren E, Charlson FJ, Patton GC, Roth GA, Hosgood HD, Whiteford HA, Kyu H, Erskine HE, Huang H, Martopullo I, Singh JA, Nachega JB, Sanabria JR, Abbas K, Ong K, Tabb K, Krohn KJ, Cornaby L, Degenhardt L, Moses M, Farvid M, Griswold M, Criqui M, Bell M, Nguyen M, Wallin M, Mirarefin M, Qorbani M, Younis M, Fullman N, Liu P, Briant P, Gona P, Havmoller R, Leung R, Kimokoti R, Bazargan-Hejazi S, Hay SI, Yadgir S, Biryukov S, Vollset SE, Alam T, Frank T, Farid T, Miller T, Vos T, Bärnighausen Till, Gebrehiwot TT, Yano Y, Al-Aly Z, Mehari A, Handal A, Kandel A, Anderson B, Biroscak B, Mozaffarian D, Dorsey ER, Ding EL, Park E, Wagner G, Hu G, Chen H, Sunshine JE, Khubchandani J, Leasher J, Leung J, Salomon J, Unutzer J, Cahill L, Cooper L, Horino M, Brauer M, Breitborde N, Hotez P, Topor-Madry R, Soneji S, Stranges S, James S, Amrock S, Jayaraman S, Patel T, Akinyemiju T, Skirbekk V, Kinfu Y, Bhutta Z, Jonas JB, Murray CJL, US Burden of Disease Collaborators (2018). The State of US Health, 1990-2016: Burden of Diseases, Injuries, and Risk Factors Among US States. J Am Med Assoc.

[2] Thompson F, Subar A (2017). Dietary assessment methodology.

[3] Wansink B (2006). Mindless eating: Why we eat more than we think.

[4] Johnson RK, Soultanakis RP, Matthews DE (1998). Literacy and body fatness are associated with underreporting of energy intake in US low-income women using the multiple-pass 24-hour recall: a doubly labeled water study. J Am Diet Assoc.

[5] Maurer J, Taren DL, Teixeira PJ, Thomson CA, Lohman TG, Going SB, Houtkooper LB (2006). The psychosocial and behavioral characteristics related to energy misreporting. Nutr Rev.

[6] Mendez MA, Popkin BM, Buckland G, Schroder H, Amiano P, Barricarte A, Huerta J, Quirós JR, Sánchez M, González CA (2011). Alternative methods of accounting for underreporting and overreporting when measuring dietary intake-obesity relations. Am J Epidemiol.

[7] Poslusna K, Ruprich J, de Vries JH, Jakubikova M, van't Veer P (2009). Misreporting of energy and micronutrient intake estimated by food records and 24 hour recalls, control and adjustment methods in practice. Br J Nutr.

[8] Subar AF, Kipnis V, Troiano RP, Midthune D, Schoeller DA, Bingham S, Sharbaugh CO, Trabulsi J, Runswick S, Ballard-Barbash R, Sunshine J, Schatzkin A (2003). Using intake biomarkers to evaluate the extent of dietary misreporting in a large sample of adults: the OPEN study. Am J Epidemiol.

[9] Shim J, Oh K, Kim HC (2014). Dietary assessment methods in epidemiologic studies. Epidemiol Health.

[10] Rhee JJ, Sampson L, Cho E, Hughes MD, Hu FB, Willett WC (2015). Comparison of methods to account for implausible reporting of energy intake in epidemiologic studies. Am J Epidemiol.

[11] Mendez MA (2015). Invited commentary: dietary misreporting as a potential source of bias in diet-disease associations: future directions in nutritional epidemiology research. Am J Epidemiol.

[12] Thompson FE, Subar AF, Loria CM, Reedy JL, Baranowski T (2010). Need for technological innovation in dietary assessment. J Am Diet Assoc.

[13] McCabe-Sellers B (2010). Advancing the art and science of dietary assessment through technology. J Am Diet Assoc.

[14] Zhu F, Bosch M, Boushey CJ, Delp EJ (2010). An image analysis system for dietary assessment and evaluation. Proc Int Conf Image Proc.

[15] Probst Y, Nguyen DT, Tran MK, Li W (2015). Dietary assessment on a mobile phone using image processing and pattern recognition techniques: algorithm design and system prototyping. Nutrients.

[16] Jia W, Yue Y, Fernstrom JD, Yao N, Sclabassi RJ, Fernstrom MH, Sun M (2012). Imaged based estimation of food volume using circular referents in dietary assessment. J Food Eng.

[17] Nelson M, Atkinson M, Darbyshire S (1996). Food photography II: use of food photographs for estimating portion size and the nutrient content of meals. Br J Nutr.

[18] Desendorf J, Bassett DR, Raynor HA, Coe DP (2014). Validity of the Bite Counter device in a controlled laboratory setting. Eat Behav.

[19] Dong Y, Hoover A, Scisco J, Muth E (2012). A new method for measuring meal intake in humans via automated wrist motion tracking. Appl Psychophysiol Biofeedback.

[20] Scisco JL, Muth ER, Hoover AW (2014). Examining the utility of a bite-count-based measure of eating activity in free-living human beings. J Acad Nutr Diet.

[21] Kalantarian H, Sarrafzadeh M (2015). Audio-based detection and evaluation of eating behavior using the smartwatch platform. Comput Biol Med.

[22] Amft O, Tröster G (2008). Recognition of dietary activity events using on-body sensors. Artif Intell Med.

[23] Fontana JM, Sazonov ES (2013). Evaluation of chewing and swallowing sensors for monitoring ingestive behavior. Sens Lett.

[24] Päßler S, Wolff M, Fischer WJ (2012). Food intake monitoring: an acoustical approach to automated food intake activity detection and classification of consumed food. Physiol Meas.

[25] Sazonov E, Schuckers S, Lopez-Meyer P, Makeyev O, Sazonova N, Melanson EL, Neuman M (2008). Non-invasive monitoring of chewing and swallowing for objective quantification of ingestive behavior. Physiol Meas.

[26] Kalantarian H, Alshurafa N, Le T, Sarrafzadeh M (2015). Monitoring eating habits using a piezoelectric sensor-based necklace. Comput Biol Med.

[27] Dong B, Biswas S (2013). Wearable diet monitoring through breathing signal analysis. Conf Proc IEEE Eng Med Biol Soc.

[28] Farooq M, Fontana JM, Sazonov E (2014). A novel approach for food intake detection using electroglottography. Physiol Meas.

[29] Farooq M, Sazonov E (2016). A novel wearable device for food intake and physical activity recognition. Sensors (Basel).

[30] Zhu F, Bosch M, Boushey CJ, Delp EJ (2010). An image analysis system for dietary assessment and evaluation. Proc Int Conf Image Proc.

[31] Chen N, Lee YY, Rabb M, Schatz B (2010). Toward dietary assessment via mobile phone video cameras. AMIA Annu Symp Proc.

[32] Daugherty BL, Schap TE, Ettienne-Gittens R, Zhu FM, Bosch M, Delp EJ, Ebert DS, Kerr DA, Boushey CJ (2012). Novel technologies for assessing dietary intake: evaluating the usability of a mobile telephone food record among adults and adolescents. J Med Internet Res.

[33] Kawano Y, Yanai K (2013). Real-time mobile food recognition system. IEEE Comput Soc Conf.

[34] Kong FY, He HS, Raynor HA, Tan JD (2015). DietCam: multi-view regular shape food recognition with a camera phone. Pervasive Mob Comput.

[35] Rahman M, Pickering M, Kerr D, Boushey C, Delp E (2012). A new texture feature for improved food recognition accuracy in a mobile phone based dietary assessment system.

[36] Shiffman S, Stone AA, Hufford MR (2008). Ecological momentary assessment. Annu Rev Clin Psychol.

[37] Liberati A, Altman DG, Tetzlaff J, Mulrow C, Gøtzsche PC, Ioannidis JPA, Clarke M, Devereaux PJ, Kleijnen J, Moher D (2009). The PRISMA statement for reporting systematic reviews and meta-analyses of studies that evaluate health care interventions: explanation and elaboration. J Clin Epidemiol.

[38] Higgins J, Green S (2008). Cochrane Handbook for Systematic Reviews of Interventions.

[39] Ashman AM, Collins CE, Brown LJ, Rae KM, Rollo ME (2017). Validation of a smartphone image-based dietary assessment method for pregnant women. Nutrients.

[40] Boushey CJ, Spoden M, Delp EJ, Zhu F, Bosch M, Ahmad Z, Shvetsov YB, DeLany JP, Kerr DA (2017). Reported energy intake accuracy compared to doubly labeled water and usability of the mobile food record among community dwelling adults. Nutrients.

[41] Bucher Della Torre S, Carrard I, Farina E, Danuser B, Kruseman M (2017). Development and evaluation of e-CA, an electronic mobile-based food record. Nutrients.

[42] Grenard JL, Stacy AW, Shiffman S, Baraldi AN, MacKinnon DP, Lockhart G, Kisbu-Sakarya Y, Boyle S, Beleva Y, Koprowski C, Ames SL, Reynolds KD (2013). Sweetened drink and snacking cues in adolescents: a study using ecological momentary assessment. Appetite.

[43] Hingle M, Yoon D, Fowler J, Kobourov S, Schneider ML, Falk D, Burd R (2013). Collection and visualization of dietary behavior and reasons for eating using Twitter. J Med Internet Res.

[44] Martin CK, Correa JB, Han H, Allen HR, Rood JC, Champagne CM, Gunturk BK, Bray GA (2012). Validity of the remote food photography method (rfpm) for estimating energy and nutrient intake in near real-time. Obesity (Silver Spring).

[45] Schüz B, Bower J, Ferguson SG (2015). Stimulus control and affect in dietary behaviours. An intensive longitudinal study. Appetite.

[46] Seto E, Hua J, Wu L, Shia V, Eom S, Wang M, Li Y (2016). Models of individual dietary behavior based on smartphone data: The influence of routine, physical activity, emotion, and food environment. PLoS One.

[47] Thomas JG, Bond DS, Ryder BA, Leahey TM, Vithiananthan S, Roye GD, Wing RR (2011). Ecological momentary assessment of recommended postoperative eating and activity behaviors. Surg Obes Relat Dis.

[48] Waki K, Fujita H, Uchimura Y, Omae K, Aramaki E, Kato S, Lee H, Kobayashi H, Kadowaki T, Ohe K (2014). Dialbetics: a novel smartphone-based self-management support system for type 2 diabetes patients. J Diabetes Sci Technol.

[49] Martin CK, Han H, Coulon SM, Allen HR, Champagne CM, Anton SD (2009). A novel method to remotely measure food intake of free-living individuals in real time: the remote food photography method. Br J Nutr.

[50] Nicklas T, Saab R, Islam NG, Wong W, Butte N, Schulin R, Liu Y, Apolzan JW, Myers CA, Martin CK (2017). Validity of the remote food photography method against doubly labeled water among minority preschoolers. Obesity (Silver Spring).

[51] Kato S, Waki K, Nakamura S, Osada S, Kobayashi H, Fujita H, Kadowaki T, Ohe K (2015). Validating the use of photos to measure dietary intake: the method used by DialBetics, a smartphone-based self-management system for diabetes patients. Diabetol Int.

[52] Seto E, Hua J, Wu L, Bestick A, Shia V, Eom S, Han J, Wang M, Li Y (2014). The Kunming CalFit study: modeling dietary behavioral patterns using smartphone data. Conf Proc IEEE Eng Med Biol Soc.

[53] Aflague TF, Boushey CJ, Guerrero RTL, Ahmad Z, Kerr DA, Delp EJ (2015). Feasibility and use of the mobile food record for capturing eating occasions among children ages 3-10 years in Guam. Nutrients.

[54] Six BL, Schap TE, Zhu FM, Mariappan A, Bosch M, Delp EJ, Ebert DS, Kerr DA, Boushey CJ (2010). Evidence-based development of a mobile telephone food record. J Am Diet Assoc.

[55] Bathgate KE, Sherriff JL, Leonard H, Dhaliwal SS, Delp EJ, Boushey CJ, Kerr DA (2017). Feasibility of assessing diet with a mobile food record for adolescents and young adults with down syndrome. Nutrients.

[56] Kerr DA, Dhaliwal SS, Pollard CM, Norman R, Wright JL, Harray AJ, Shoneye CL, Solah VA, Hunt WJ, Zhu F, Delp EJ, Boushey CJ (2017). BMI is associated with the willingness to record diet with a mobile food record among adults participating in dietary interventions. Nutrients.

[57] Kerr DA, Harray AJ, Pollard CM, Dhaliwal SS, Delp EJ, Howat PA, Pickering MR, Ahmad Z, Meng X, Pratt IS, Wright JL, Kerr KR, Boushey CJ (2016). The connecting health and technology study: a 6-month randomized controlled trial to improve nutrition behaviours using a mobile food record and text messaging support in young adults. Int J Behav Nutr Phys Act.

[58] Kerr DA, Pollard CM, Howat P, Delp EJ, Pickering M, Kerr KR, Dhaliwal SS, Pratt IS, Wright J, Boushey CJ (2012). Connecting health and technology (chat): Protocol of a randomized controlled trial to improve nutrition behaviours using mobile devices and tailored text messaging in young adults. BMC Public Health.

[59] Elliston KG, Ferguson SG, Schüz N, Schüz B (2017). Situational cues and momentary food environment predict everyday eating behavior in adults with overweight and obesity. Health Psychol.

[60] Waki K, Aizawa K, Kato S, Fujita H, Lee H, Kobayashi H, Ogawa M, Mouri K, Kadowaki T, Ohe K (2015). Dialbetics with a multimedia food recording tool, foodlog: smartphone-based self-management for type 2 diabetes. J Diabetes Sci Technol.

[61] Berkman ET, Giuliani NR, Pruitt AK (2014). Comparison of text messaging and paper-and-pencil for ecological momentary assessment of food craving and intake. Appetite.

[62] Bruening M, Ohri-Vachaspati P, Brewis A, Laska M, Todd M, Hruschka D, Schaefer DR, Whisner CM, Dunton G (2016). Longitudinal social networks impacts on weight and weight-related behaviors assessed using mobile-based ecological momentary assessments: study protocols for the SPARC study. BMC Public Health.

[63] Dunton GF, Liao Y, Dzubur E, Leventhal AM, Huh J, Gruenewald T, Margolin G, Koprowski C, Tate E, Intille S (2015). Investigating within-day and longitudinal effects of maternal stress on children's physical activity, dietary intake, and body composition: protocol for the MATCH study. Contemp Clin Trials.

[64] Miller CK, Weinhold KR, Mitchell DC (2016). Using ecological momentary assessment to track goal progress toward the adoption of a low glycemic index diet among adults with type 2 diabetes: a pilot study. Top Clin Nutr.

[65] Powell DJ, McMinn D, Allan JL (2017). Does real time variability in inhibitory control drive snacking behavior? An intensive longitudinal study. Health Psychol.

[66] Richard A, Meule A, Reichenberger J, Blechert J (2017). Food cravings in everyday life: an EMA study on snack-related thoughts, cravings, and consumption. Appetite.

[67] Spook JE, Paulussen T, Kok G, Van Empelen P (2013). Monitoring dietary intake and physical activity electronically: feasibility, usability, and ecological validity of a mobile-based Ecological Momentary Assessment tool. J Med Internet Res.

[68] Strahler J, Nater UM (2018). Differential effects of eating and drinking on wellbeing-an ecological ambulatory assessment study. Biol Psychol.

[69] Wouters S, Thewissen V, Duif M, Lechner L, Jacobs N (2016). Assessing energy intake in daily lifeignal-contingent smartphone application versus event-contingent paper and pencil estimated diet diary. Psychologica Belgica.

[70] Zenk SN, Horoi I, McDonald A, Corte C, Riley B, Odoms-Young AM (2014). Ecological momentary assessment of environmental and personal factors and snack food intake in African American women. Appetite.

[71] Bruening M, van Woerden I, Todd M, Brennhofer S, Laska MN, Dunton G (2016). A mobile ecological momentary assessment tool (devilsparc) for nutrition and physical activity behaviors in college students: a validation study. J Med Internet Res.

[72] Liao Y, Schembre SM, O'Connor SG, Belcher BR, Maher JP, Dzubur E, Dunton GF (2018). An electronic ecological momentary assessment study to examine the consumption of high-fat/high-sugar foods, fruits/vegetables, and affective states among women. J Nutr Educ Behav.

[73] O'Connor SG, Koprowski C, Dzubur E, Leventhal AM, Huh J, Dunton GF (2017). Differences in mothers' and children's dietary intake during physical and sedentary activities: an ecological momentary assessment study. J Acad Nutr Diet.

[74] O'Connor SG, Ke W, Dzubur E, Schembre S, Dunton GF (2018). Concordance and predictors of concordance of children's dietary intake as reported via ecological momentary assessment and 24 h recall. Public Health Nutr.

[75] Wouters S, Jacobs N, Duif M, Lechner L, Thewissen V (2018). Affect and between-meal snacking in daily life: the moderating role of gender and age. Psychol Health.

[76] McMinn D, Allan JL (2014). The SNAPSHOT study protocol: SNAcking, Physical activity, Self-regulation, and Heart rate Over Time. BMC Public Health.

[77] Shiffman S, Stone AA, Shiffman S, Atienza AA, Nebling L (2007). Designing protocols for ecological momentary assessment. The Science of Real-Time Data Capture: Self-Reports in Health Research.

[78] Kampmeijer R, Pavlova M, Tambor M, Golinowska S, Groot W (2016). The use of e-health and m-health tools in health promotion and primary prevention among older adults: a systematic literature review. BMC Health Serv Res.

[79] Tennant B, Stellefson M, Dodd V, Chaney B, Chaney D, Paige S, Alber J (2015). eHealth literacy and Web 2.0 health information seeking behaviors among baby boomers and older adults. J Med Internet Res.

[80] Stone AA, Shiffman S (2002). Capturing momentary, self-report data: a proposal for reporting guidelines. Ann Behav Med.

[81] Timon CM, van der Berg R, Blain RJ, Kehoe L, Evans K, Walton J, Flynn A, Gibney ER (2016). A review of the design and validation of web- and computer-based 24-h dietary recall tools. Nutr Res Rev.

