# Supplementary material for: Mobile Ecological Momentary Diet Assessment Methods for Behavioral Research: Systematic Review
Source: JMIR Mhealth Uhealth. 2018 Nov 20;6(11):e11170. doi: 10.2196/11170 (PMC6280032; doi:10.2196/11170)
Supplement: Multimedia Appendix 1 [file mhealth_v6i11e11170_app1.pdf]

Multimedia Appendix 1. Data Extraction List

- First Author
- Year
- Title
- Study name
- Goal of Research
- Population(s) studied
- Mobile device
- Operating System
- Training paradigm
- Duration of sampling
- Sampling contingency (event, signal, etc.)
- Frequency of sampling
- Prompt notification (if applicable)
- Prompt Scheduling (Prompt frequency and times, if applicable)
- Data collection timing (Real time or retrospective)
- Data entry restrictions and reminders
- Valid response rate (compliance, if applicable)
- Diet data (source)
- Diet data collection (response format)
- Dietary variable source
- Level of diet data output
- Diet data output (units)
- Concurrent data collected
- Additional details provided in:
-
